# Wharton’s Jelly-Derived Mesenchymal Stromal Cells as a Promising Cellular Therapeutic Strategy for the Management of Graft-versus-Host Disease

**DOI:** 10.3390/ph8020196

**Published:** 2015-04-16

**Authors:** Joseph P. McGuirk, J. Robert Smith, Clint L. Divine, Micheal Zuniga, Mark L. Weiss

**Affiliations:** 1 Blood and Marrow Transplant Program, The University of Kansas Medical Center, 2330 Shawnee Mission Pkwy., Suite 210 Mailstop 5003, Westwood, KS 66205, USA; 2 Department of Anatomy and Physiology, Kansas State University, 1600 Denison Ave., Coles Hall 228, Manhattan, KS 66506-5802, USA; E-Mails: robs32@vet.k-state.edu (J.R.S.); mjzuniga@k-state.edu (M.Z.); weiss@vet.k-state.edu (M.L.W.)

**Keywords:** allo-HCT, GVHD, WJ-MSCs

## Abstract

Allogeneic hematopoietic cell transplantation (allo-HCT), a treatment option in hematologic malignancies and bone marrow failure syndromes, is frequently complicated by Graft-versus-host disease (GVHD). The primary treatment for GVHD involves immune suppression by glucocorticoids. However, patients are often refractory to the steroid therapy, and this results in a poor prognosis. Therefore alternative therapies are needed to treat GVHD. Here, we review data supporting the clinical investigation of a novel cellular therapy using Wharton’s jelly (WJ)-derived mesenchymal stromal cells (MSCs) as a potentially safe and effective therapeutic strategy in the management of GVHD. Adult-derived sources of MSCs have demonstrated signals of efficacy in the management of GVHD. However, there are limitations, including: limited proliferation capacity; heterogeneity of cell sources; lengthy expansion time to clinical dose; expansion failure *in vitro*; and a painful, invasive, isolation procedure for the donor. Therefore, alternative MSC sources for cellular therapy are sought. The reviewed data suggests MSCs derived from WJ may be a safe and effective cellular therapy for GVHD. Laboratories investigated and defined the immune properties of WJ-MSCs for potential use in cellular therapy. These cells represent a more uniform cell population than bone marrow-derived MSCs, displaying robust immunosuppressive properties and lacking significant immunogenicity. They can be collected safely and painlessly from individuals at birth, rapidly expanded and stored cryogenically for later clinical use. Additionally, data we reviewed suggested licensing MSCs (activating MSCs by exposure to cytokines) to enhance effectiveness in treating GVHD. Therefore, WJCs should be tested as a second generation, relatively homogeneous allogeneic cell therapy for the treatment of GVHD.

## 1. Introduction: Allogeneic Hematopoietic Cell Transplantation and Acute Graft-versus-Host Disease

Allogeneic hematopoietic cell transplantations (allo-HCT) are increasingly used as a treatment for management of hematologic malignancies, bone marrow failure syndromes, and inborn errors of metabolism [1]. They are often complicated by graft-versus-host disease (GVHD), a common cause of non-relapse morbidity and mortality. The curative potential for allo-HCT, when applied as a therapy in the management of hematologic malignancies, specifically derives from an immunologically driven, graft-versus-tumor effect mediated principally by donor T cells, and associated with a lesser risk for relapse when compared to high-dose chemo-radio therapy and autologous HCT. Donor-derived T cells are also responsible for mediating the occurrence of GVHD, a common transplant-related complication affecting a significant percentage of patients undergoing allo-HCT [2,3,4]. Second only to relapse of the primary disease for which the transplant was employed as a cause of morbidity and mortality following allo-HCT, GVHD represents the major non-relapse barrier to the success of this otherwise potentially curative treatment [2].

GVHD can be classified as an acute (aGVHD) or chronic (cGVHD) clinical syndrome. Both have distinct clinical manifestations, natural histories, treatment responses and prognoses, although overlapping presentations are relatively common [5,6]. Acute GVHD manifests commonly as an acute inflammatory process primarily involving the integument, intestinal tract, liver, and frequently presents as a maculopapular rash, nausea, vomiting, diarrhea and hepatic cholestasis. In contrast, the cGVHD’s inflammation leads to fibrosis of involved organs and frequently presents clinically with sicca syndrome-like features, scleradermatous-like skin changes, cytopenias, and chronic fibrosing pulmonary, hepatic and intestinal manifestations. The severity of aGVHD can be determined by a staging/grading system [7]. Patients with aGVHD grades I−II experience five-year leukemia-free survival of 44%–51%; in contrast, survival decreases to 26% for patients with grade III and 7% for grade IV aGVHD [8]. The severity of chronic GVHD historically could be determined as either limited or extensive, although recently a classification schema has improved prognostication and treatment response determinations [9].

## 2. Current Risk Factors and Disadvantages with Graft-versus-Host Disease (GVHD)

Chronic GVHD associates with a decreased risk of relapse of hematologic malignancies after allo-HCT. However, it continues to represent the leading cause of late treatment-related deaths among allo-HCT recipients. GVHD commonly targets the thymus, resulting in distortion and disruption of normal thymic architecture, thereby leading to defective thymopoiesis. Both acute and chronic forms impact thymic recovery after allo-HCT, and this associates with reduction in naïve T cells and T-cell receptor excision circles (TRECs), which results in a narrow T-cell repertoire. This fact, in conjunction with a necessary escalation in the immunosuppressive drugs used to treat GVHD, results in increased systemic infections and significant associated morbidity and mortality [10,11,12].

The risk factors associated with the development and severity of GVHD include human leukocyte antigen (HLA) mismatching between donor and recipient, sex mismatching, advanced recipient and/or donor age, stem-cell source, and methodology of GVHD prophylaxis [13,14]. GVHD commonly occurs in patients undergoing HLA-matched sibling and unrelated donor transplants. This mismatching likely can be attributed to donor-recipient mismatches for minor histocompatibility antigens not currently accounted for in routine HLA typing [15,16].

Importantly, the majority of patients that allo-HCT could benefit does not have an HLA-matched sibling donor. This problem was addressed by developing registries for HLA-matched unrelated donors, which facilitated access to allogeneic stem-cell transplantation for many patients. However, significant under-representation for many ethnic groups exists in current registries [17]. Therefore, alternate graft sources have increasingly been utilized. For patients who lack HLA-identical siblings or unrelated donors, development of novel therapeutic strategies has led to remarkable growth in the use of HLA mismatched unrelated adult and cord blood stem cell sources, as well as haploidentical related donors. The increased use of alternative donor stem cells in allo-HCT accompanies an increased risk for transplant-related complications and requires more effective prophylactic and treatment strategies [18].

Currently, prophylactic strategies are most effective at contending with the risk of acute and chronic GVHD. Although many prophylactic strategies have been studied, the most commonly employed are optimal HLA matching at MHC class I and II loci between donor and recipient, pharmacologic approaches that are employed to block T-cell antigen recognition and resultant proliferation during the early initiating phases of aGVHD. The most commonly employed pharmacologic strategies consist of a calcineurin inhibitor in combination with methotrexate or mycophenolate mofetil and an mechanistic target of rapamycin (MTOR) inhibitor such as rapamycin [19]. Approaches that are less common, but increasingly in use, include graft manipulation through *in vivo* or *ex vivo* T-cell depletion strategies and limiting tissue damage caused by the preparative regimen through the employment of less intensive, yet adequately immunosuppressive chemo-radiation regimens. Despite these prophylactic measures, often inflammatory cascades are triggered and donor T cells begin destroying host tissues.

The standard initial treatment for both acute and chronic GVHD is steroid-based therapy. Unfortunately, significant percentages of patients become resistant to steroid therapy and subsequently must be treated with second-line immunosuppressive agents [5,6]. Steroid-refractory aGVHD portends a poor prognosis; second-line agents frequently prove ineffective and, as a result, survival is <10% at five years. Therefore, alternative therapies are needed to treat GVHD following allo-HCT, particularly in the setting of steroid-resistant disease.

## 3. Advantages of Bone Marrow-Derived Mesenchymal Stromal Cells (MSCs) as Cellular Therapy for Acute Graft-versus-Host Disease (aGHVD)

Many immunosuppressive strategies have been studied for steroid-refractory aGVHD, though none have proven to be consistently effective and safe for this clinical problem. Promising treatments for steroid-refractory aGVHD involve the infusion of third-party, HLA-disparate, unrelated bone marrow derived mesenchymal stromal cells (BM-MSC). The *in vivo* and *in vitro* properties of BM-MSC suggest their potential use in a broad range of inflammatory and immune-mediated conditions, such as GVHD. BM-MSC are a population of undifferentiated multipotent mesenchymal stromal cells which express HLA class I and do not express HLA class II or costimulatory molecules CD40, CD80 or CD86 [20,21,22,23]. BM-MSC have been demonstrated to modulate immune and inflammatory responses in animal models of inflammatory disease including GVHD [24,25,26,27,28], and to facilitate repair of connective tissues [29,30,31,32].

MSCs inhibit T cells that have been induced by a variety of stimuli from activating and proliferating [23,33]. They also down-regulate inflammatory cytokine expression such as tumor necrosis factor (TNF)-α, IL2R-α, elafin, and interferon-γ (IFN-γ) [34,35]. Dander *et al.* investigated the effects of MSC infusion on lymphocyte counts in transplanted patients with steroid-refractory GVHD [35]. They found CD4^+^ T cell subsets changed significantly after MSC infusion and significant improvement in patient symptoms was associated with an increase in Tregs increased and decrease in Th1 and Th17 Le Blanc *et al.* [36] reported the first case of successful treatment for severe refractory aGVHD using *ex vivo* expanded haploidentical MSCs. Their subsequent report demonstrated a positive therapeutic effect using allogeneic MSCs in patients experiencing steroid-refractory aGVHD with no significant adverse events attributed to the cells [37].

After the initial reports of safety and tolerance, additional studies reported encouraging clinical results and confirmed the safety of MSCs in the treatment of steroid-refractory aGVHD [36,37,38,39,40,41,42,43,44,45,46,47,48,49,50,51,52,53,54,55,56,57,58]. Illustrative demonstrations for efficacy and safety have been reported to date by multiple investigators. Kurtzberg *et al.* reported [59] that using allogeneic MSCs as a rescue agent for severe treatment-resistant aGVHD demonstrated a 64% response rate in 59 children by day 28, and the response to MSCs correlated with improved overall survival at 100 days [45]. This work suggests an excellent risk/benefit profile for MSC therapy [45,60]. Martin *et al.* reported a randomized, placebo-controlled, multi-center phase III trial of MSCs in the treatment of steroid-refractory aGVHD involving 244 patients [48]. Although the endpoint of durable complete response >28 days was not significantly better in the MSC-treated population, significant differences in response for patients with multi-organ involvement, liver and intestinal involvement were found for the MSC-treated cohort.

Table 1 summarizes the published reports describing the clinical outcomes for patients treated with MSCs in the management of both aGVHD and cGVHD [36,37,38,39,40,41,42,43,44,45,46,47,48,49,50,51,52,53,54,55,56,57,58]. These reports included patients that received a variety of conditioning regimens including myeloablative, or non-myeloablative, or reduced intensity conditioning (RIC), with no apparent differences in the response to MSC treatment. Furthermore, patients included in these reports received MSCs from many sources including HLA-identical, haploidentical, or third party, unrelated and unmatched donors. The majority of clinical data reported used BM-MSCs; however, other sources of MSCs have been studied. Fang *et al.* used MSCs derived from adipose tissue [40,41,42], with no apparent differences in response or safety compared to BM-MSCs. Important for the availability of off-the-shelf cell therapy, MSCs from freshly expanded samples or from cryogenically stored/thawed cell preparations have been used as well, with no apparent differences in response [61]. MSCs have been shown to be safe: no ectopic tissue formation has been derived from infused MSCs in animal models or human studies. [62,63]. Finally, MSCs caused no harm: no clearly defined increased incidence of opportunistic infections or relapse of malignancy have been reported to date [64]. In summary, the data support the concept of MSCs as a safe, well-tolerated and variably effective treatment for GVHD. Importantly, MSCs can be cryogenically banked, thawed and given without the need for donor-recipient HLA-matching.

## 4. Limitations of Bone Marrow-Derived MSCs as a Cellular Therapy for aGVHD

There are specific problems that limit BM-MSCs usefulness. The isolation requires aspiration from the marrow cavity which is a painful, invasive procedure, with certain risks. Several studies demonstrate a limited expansion potential or slower expansion for adult-derived MSCs *in vitro* compared to fetal tissue-derived MSCs. Furthermore, adult MSCs may be less-responsive than fetal or neonatal MSCs in certain applications [65,66,67,68,69,70]. Therefore, alternative tissue sources, such as discarded tissues resulting from pregnancy, containing fetal-derived MSCs have been considered as an alternative MSC source. Recently, an exploratory clinical study using fetal-derived MSCs was reported demonstrating feasibility, safety and efficacy [56]. This review focuses on two areas related to the potential for these tissues, with the aim to improve the next iteration of clinical trials. First, we review the literature suggesting that MSCs from discarded fetal tissues might offer certain advantages over BM-MSCs for GVHD therapy. This idea relates to BM-MSCs’ aforementioned limitations that may be overcome using an alternative MSC source. Second, we reviewed literature suggesting *in vitro* conditioning by cytokine exposure, called “licensing,” of MSCs during expansion could improve their clinical effect in GVHD. This subject relates to the relative plasticity of MSCs to culture conditions such as hypoxia, cytokine exposure, *etc.*, that changes the MSC physiology and may improve their clinical effect. Lastly, we review MSCs’ impact on immunophysiology during GVHD. We theorize that MSCs derived from the umbilical cord may be an effective alternate source for MSCs, which are safely and painlessly collected to replace BM-MSC, for GVHD prevention or treatment.

## 5. Possible Advantages of Umbilical Cord-Derived MSCs

The stroma of umbilical cord, also known as Wharton’s jelly (WJ, or WJCs below), is a source of primitive, loose connective tissue that is rich in hyaluronan. WJ supports and cushions the umbilical vessels. WJ contains an MSC population. The population of MSC in WJ has certain advantages to other MSC sources (see Table 2). For example, it is easily, safely and painlessly harvested following birth from the discarded umbilical cord after the umbilical cord blood has been collected. WJCs grow more quickly and produce more cells during expansion *in vitro* compared with BM-MSCs [71,72] and they have immune properties similar to adult-derived MSCs from bone marrow and adipose tissue [68,71,73,74,75].

**Table 1:** GVHD patients treated with MSCs.

Citation	MSC Source	MSC Donor	No. of Patients/GVHD Grade	HCT Conditioning	Dose of MSCs (/kg)	Effect on GVHD	Ref.
LeBlanc, K. *et al.*, 2004	BM	Haploidentical (mother)	1 (9 yr boy) Grade 4	Myeloablative	2 × 10^6^ first; 1 × 10^6^ second	Improvements	33
Ringden, O. *et al.*, 2006	BM	HLA identical sib n = 2; Haploidentical n = 6; HLA mismatch n = 4	9 (12 infusions); steroid refractory; aGVHD 8; cGVHD 1	Myeloablative n = 5; RIC n = 3; ATG only n = 4	0.6–9 × 10^6^	Complete response 6; Response 1; Slight effect 1; No response 4	34
Fang, B. *et al*. 2006, 2006, 2007, 2009	Adipose MSC	Unrelated mismatch; Haploidnetical; Haploidentical; Unrelated mismatch n = 4	1 (38 yr); 2 steroid refractory; 1 chronic hepatic; 6-steroid refractory aGVHD	Myeloablative	2 × 10^6^ first; 1 × 10^6^ second	Complete response; Complete response; Complete response; Complete response; 5/6 complete response	37–40
Muller, I. *et al.*, 2008	BM	Mismatch family n = 8; HLA identical n = 2; HLA matched unrelated n = 1	7 (11 infusions); aGVHD n = 2; cGVHD n = 3; Hemophagocytosis n = 1; Graft rejection prophylaxis n = 1	Myeloablative n = 5; RIC n = 2	0.4–3 × 10^6^	aGVHD 1/2 alive and well; cGVHD 1/3 slide improvement; Hemophagocytosis, good response; Graft rejection prophylaxis alive and well	46
LeBlanc, K. *et al.*, 2008	BM	HLA identical sib n = 5; Haploidentical n = 18; Unrelated mismatch n = 69	55 (92 infusions); Grade 2 n = 5; Grade 3 n = 25 Grade 4 n = 25	Unknown	0.4–9 × 10^6^	Children 17/25 complete response, 4/25 partial response Adult 13/30 complete response, 5/30 partial response; total 30/55 complete response (54%), partial response 9/55 (16%) Overall 2 yr survival 53% for complete response vs 16% for partial or non-response	43
von Bonin, M. *et al.*, 2008	BM	Unrelated mismatched	13 (32 infusions) Grade 3 n = 2 Grade 4 n = 11	Myeloablative n = 1 RIC n = 12	0.6 × 10^6^ (0.6–1.1)	2 patients (15%) complete response 5/11 (45%) partial response	47
Zhou, H. *et al.*, 2009	BM	HLA matched, unrelated	4 cGVHD	Nonmyeloablative n = 4	1–2 × 10^7^ (4 to 8 infusions)	4/4 Complete response	49
Kebriaei, P. *et al.*, 2009	BM	Osiris unrelated unmatched n = 6	31 (62 infusions) Grade 2 n = 21 Grade 3 n = 7 Grade 4 n = 3	Myeloablative n = 15 RIC n = 8 Nonmyloablative n = 4 DLI n = 4	2 × 106 n = 16 8 × 106 n = 15	24/31 Complete response, 5 partial response 2 No response	41
Arima, N. *et al.*, 2010	BM	Related, HLA identical n = 1 Unknown	3 Grade 3 n = 3	RIC n = 1 unknown n = 2	0.5–2 × 10^6^ intra-arterial injection into GVHD sites	1/3 partial response	35
Baron F. *et al.*, 2010	BM	third party, mismatch	20 patients (19 historic controls)	Nonmyeloablative, coinfusion w/ MSC n = 20	Unknown	MSC coinfusion appears safe MSC coninfusion might prevent death from GVHD without impacting GVT	36
Lucchini, G. *et al.*, 2010	BM	Single donor unrelated HLA mismatch	aGVHD Grade 1–4 or cGVHD n = 11	Variable: TBI, RIC, *etc.*	0.7–3.7 × 10^6^ 1–5 infusions	8/11 Complete (23%) or partical (47%) response 3 No response	44
Weng, J.Y. *et al.*, 2010	BM	HLA matched third party, mismatched	cGVHD 73% severe, 26% moderate) n = 19	Variable: TBI, RIC, *etc.*	0.23–1.42 × 10^6^ 1–5 infusions	14/19 Complete (4) or partial (10) response 5 Died	48
Sanchez-Guijo, F. *et al.*, 2014	BM	Unknown, Assume unrelated mismatch	aGVHD Grade 2–4 n = 25	unknown	0.7–1.31 × 10^6^ 4 infusions	17/24 responded 11/17 complete and 6/17 partial 7/24 no response	54
Introna, M. *et al.*, 2014	BM	Unrelated third party	GVHD, n = 40: 15 children, 25 adults	Variable: myeloablative, RIC	1.5 × 10^6^ (0.8–3.1 × 10^6^) Median of 3 infusions (range 2–7 infusions)	27/40 responded, 11/40 complete, 16/40 partial. Children and adults responded at same rate. 18 Died	51
Resnick, I.B. *et al.*, 2013	BM	Third party fully mismatched (62), third party haploidentical (5)	aGVHD, grade 2–4, n = 50	Myeloablative (45), RIC (5); TBI (13) and fludarabine (32)	1.14 × 10^6^ (0.3–4.27 range) Range of 1–4 infusions per patient	17/50 complete, 33/50 partial response. 5 Died	52
Ball, L.M. *et al.*, 2013	BM	Unrelated third party	aGVHD, grade 3–4, n = 37 children, median age 7	Variable: 9 TBI, 28 chemotherapy-based	1–2 × 10^6^ Median of 2 infusions (range 1–13 infusions)	24/37 complete, 13/37 partial, 5 no response	50
Ringden, O. *et al.*, 2013	Fetal membrane cells	Unrelated third party	aGVHD, grade 3–4, n = 9	Chemotherapy, or chemotherapy and TBI	0.9–2.8 × 10^6^	2/8 complete, 4/8 partial, 3 no response	53
Wu, K.H. *et al.*, 2011	UC-MSC	Unrelated third party	aGVHD, grade 3–4, n = 2	Chemotherapy, or chemotherapy and TBI	3.3–8.0 × 10^6^ –3 infusions	2/2 complete	55
Prasad, V.K. *et al.*, 2011	BM						
Kurtzberg, J. *et al.*, 2014	BM	Unrelated third party (Osiris)	aGVHD, grade 3–4, n = 12	11 myeloablative, 1 RIC	8 × 10^6^, Median of 8 infusion (2–21 range)	7/12 complete, 2/12 partial, 9/12 complete resolution of GI problems	42
Martin, P.J. *et al.* 2010	BM	Unrelated third party (Osiris)	GHVD, grades B–D, n = 244	unknown	2 × 10^6^, 8 infusions	76% complete or partial response at 100 day, most effective for GI and liver	45

**Table 2:** Comparison of WJ- MSC features with other MSCs.

	WJ-MSCs	BM-MSCs	AD-MSCS
**Collection**	Non-Invasive	Invasive	Invasive
**Induced Pain**	No Pain	Painful Procedure	Minimum to Moderate
**Risk of Collection**	No Risk	Moderate	Minimum to Moderate
**Source**	Fetal Origin	Adult Origin	Adult Origin
***In vitro* Expansion**	High Expansion	Moderate Expansion	Moderate Expansion
***In vitro* Growth**	Faster Growth	Slower Growth	Faster Growth

## 6. Attractiveness of MSCs

The immune properties of MSCs make them attractive for immunological disorders. These immune properties are: (1) low immunogenicity and naïve MSCs do not strongly stimulate allogeneic T-lymphocyte proliferation; (2) MSCs suppress the proliferation of activated T lymphocytes; (3) increased production of regulatory T cells; and (4) a shift in the immune response towards tolerance or anergy since MSCs do not stimulate B cells and prevent B cells from becoming stimulated.

## 7. Mechanisms for MSC Immune Suppression

The mechanisms of MSC immune suppression have been reviewed elsewhere (see [34,76,77]). GVHD may be modeled *in vitro* since treatments that impact the inflammatory response are reflected by assays of the suppression of mitogen-activated or allo-antigen-activated T-cell proliferation, as well as the expansion of regulatory T cells, which would reflect a critical component of tolerance induction. The mechanisms used by MSCs are under debate. Evidence exists to support both a direct, contact-dependent mechanism that is mediated at least in part by MSC expression of the cell death ligand, B7-H1 [78], and an indirect, non-contact dependent mechanism mediated by various cytokines and growth factors such as prostaglandin E2 (PGE2), cyclooxygenase (COX) 1 and 2, hepatocyte growth factor (HGF), transforming growth factor-β, interleukin 10, human leukocyte antigens G5 and E, leukemia inhibitor factor, indoleamine 2,3-dioxygenase (IDO), and others [33,34,74,76,77,79,80,81]. With regards to the direct mechanism, recent work indicates that a transfer of cytosolic contents from MSCs to other cells may mediate part of the MSC’s regenerative effects. This connection has also referred to as nanotubes [82,83]. As seen below, the mechanisms are not fully determined and the literature is filled with examples and counterexamples.

## 8. Comparison of MSC Immune Properties

Several studies compared the immune properties of BM-MSCs, WJCs and MSCs derived from adipose tissues [68,71,73,74,84,85]. Najar *et al.* reported that adipose-derived MSCs and WJCs had similar *in vitro* immunosuppressive effects for lymphocyte proliferation, compared to BM-MSCs: MSCs target CD4+ and CD8+ T cells for immune suppression equally; adipose-derived and WJCs inhibit T-cell activation; and MSCs were immunosuppressive regardless of the type of stimuli used to activate the lymphocytes [85]. MSC immune suppression was mediated by COX 1 and 2 enzymes and by the production of PGE2 and did not involve HGF. In agreement with Najar *et al.*’s findings, Chen *et al.* found that PGE2 synthesis, mediated by COX2, produces the majority of WJCs’ suppressive effects on T-cell proliferation and on IFN-γ secretion [65]. PGE2 expression by WJCs was stimulated by inflammatory signals IFN-γ or interleukin-1β produced by peripheral blood mononuclear cells following mitogen or allogeneic stimulation. Critically, they found that WJCs cultured with unstimulated (naïve) T cells do not secrete much PGE2; however, following co-culture with stimulated T cells, WJCs excreted more PGE2.

## 9. MSC Stimulation and Expression

MSCs have little effect on unstimulated T cells, and exposure to activated T cells or inflammatory cytokines changes MSCs so they display immunosuppressive behavior [28,68,71,74,78,86,87]. This has been termed “licensing” or “priming” of MSCs. Chen *et al.* found that indoleamine 2,3-dioxygenase (IDO) and transforming growth factor beta (TGF-β) played little role in MSC’s suppression of the T-cell proliferation. As a counterexample to Chen *et al.*’s finding that IDO had little role. Yoo *et al.* had diametrically different findings when they compared the immunoregulatory properties of adipose-derived, umbilical cord blood-derived MSCs, WJCs and BM-MSC [68]. They found that MSCs from all four tissue sources responded to either IFN-γ or tumor necrosis factor-α (TNF-α) secreted from activated T cells by inducing IDO secretion, and the released IDO from MSCs suppressed T-cell proliferation, and led to decreases in TNF-α and IFN-γ. Yoo *et al.* reported that, while MSCs responded to IFN-γ or TNF-α exposure to upregulate IDO expression, they did not increase expression of HGF, Cox 1 and 2, interleukin-10, and TGF-β. Prasanna *et al.* also examined the immune properties of MSCs from BM and WJ, in addition to the effect of IFN-γ and TNF-α exposure on these properties [74]. They found that IFN-γ or TNF-α stimulation produced subtly different responses between BM-MSCs and WJCs. For example, IFN-γ or TNF-α exposure increased the expression of the immune-adhesive ligand, CD54, in both BM-MSCs and WJCs. However, IFN-γ increased expression of HLA class 2 in BM-MSCs and not in WJCs. Prasanna *et al.* also reported that IFN-γ exposure did not strongly affect the immunogenicity of MSCs in their *in vitro* T-cell proliferation assays [74]. In contrast to these findings, Cho *et al.* reported that IFN-γ exposure induced expression of MHC class II in swine and human WJCs, and that IFN-γ stimulated WJCS produced an antibody response following subcutaneous or intravenous injection of allogeneic WJCs faster than when unlicensed WJCs were used in a swine model [86]. One unexpected finding in the Prasanna *et al.* report was the importance of MSC proliferation (possibly) on immune-suppressive properties: both BM-MSCs and WJCs that had been mitotically inactivated lost their immune suppressive effect. To our knowledge, this was the first report to correlate MSC proliferation with suppression of lymphocyte proliferation. The mechanism that makes cells with higher mitotitic index more immune suppressive is unknown. If MSC proliferation is critical for immune modulation, this would significantly impact upon how MSCs are derived for therapeutic use.

The differences between Cho *et al.* and Prasanna *et al.* on changes in HLA expression are explained by work from Deuse *et al.*, who compared the immunogenicity of allogeneic BM-MSCs and WJCs both *in vitro* and *in vivo* following exposure to different doses of IFN-γ [71]. At doses of IFN-γ below 50 ng/mL, IFN-γ upregulated HLA-DR and doses from 100–500 ng/mL of IFN-γ downregulated HLA-DR. Interestingly, in all cases, WJCs had lower expression of HLA-I and HLA-DR compared to BM-MSCs, WJCs had weaker allogeneic T-cell stimulation compared to BM-MSCs, and WJCs had longer survival following allogeneic transplantation in immunocompetent Balb/c mice.

## 10. MSCs Synthesize Anti-Inflammatory Protein Galectin

Using reverse transcriptase PCR, galectins (Gal) 1, 3, 8 and 9 have recently been identified as being produced by MSCs [88]. Gal-1 was identified as the initial lectin to be involved in the process of MSC-mediated suppression of allo-reactive T cells [89]. Knockdown of Gal-3 demonstrated that Gal-3 also plays a role in MSCs’ immunosuppressive effects on T cells [90]. The secretion of Gal-3 into the media is sufficient to cause inhibition of T-cell expansion [88]. Since Gal-3 and Gal-1 both demonstrate immunosuppressive potential, these galectins have been proposed as *in vitro* biomarkers for MSC immunomodulatory potency [88,89,91].

## 11. MSCs Synthesize Anti-Inflammatory Protein Tumor Necrosis Factor-α-Stimulated Gene 6 (TSG-6)

MSCs have been demonstrated to secrete tumor necrosis factor-α-stimulated gene 6 (TSG-6) when exposed to inflammatory stimuli [92]. TSG-6 is a hyaluronan receptor (CD44)-binding protein that inhibits TNF-α and neutrophil accumulation, and thus blocks inflammatory tissue damage [93,94]. Work from Dr. Prockop’s lab indicates that MSC homing to sites of injury is unnecessary for the MSCs’ therapeutic effect. For example, MSCs reduce inflammatory damage without homing to the site of damage (e.g., while MSCs are trapped in lungs following IV infusion) via MSC-secreted TSG-6 [95,96,97]. This leads to healing in corneal models, recovery of damaged tissue, and prevents allogeneic corneal transplantation rejection [96,98]. In diabetic models, TSG-6 was shown to inhibit Th1 responses preventing pre-diabetic mice from becoming fully diabetic [94]. In neuro-inflammation and traumatic brain injury models, the anti-inflammatory effects from MSCs by TSG-6 were mediated by downstream signaling through nuclear factor-kB and mitogen-activated protein kinases (MAPK) [99,100]. Importantly, MSCs do not need to be given at the site of injury; i.v. administration was effective and TSG-6 by itself could duplicate the effects of MSCs [94,97,98,100]. Dr. Prockop’s lab described novel 3D culture conditions that enhance MSC anti-inflammatory properties via enhanced TSG-6 and PGE2 release [92,101].

## 12. Microvesicles

As reviewed [102,103,104,105,106,107], microvesicles or a subset of microvesicles called exosomes mediate some of the therapeutic effects of MSCs. MSCs release factors into the medium, such as TSG-6, which have anti-inflammatory properties and which account for MSC’s paracrine effects [104,108]. Others have indicated that microvesicles alone are not as effective as the cellular counterpart, suggesting direct contact is important for immune modulation [109]. MSC microvesicles have been suggested as a therapy for GVHD [110]. Since MSCs are amenable to targeted modifications that may optimize them as microvesicle-delivery vehicles [106], the future may offer customized MSC-derived microvesicle formulations for off-the-shelf use for GVHD, myocardial infarction or cancer therapy from the spent medium of bioreactors [106,111].

## 13. Dendritic Cells

Since we wrote the previous review on this subject [112], the effect of MSCs on dendritic cells (DC) has been better defined. The consensus was that MSCs prevent DC maturation and inhibit cytokine production by DCs [77]. In 2011, Chiesa *et al.* showed that following intravenous injection of MSCs, DCs stop migrating to peripheral lymph nodes within 20 minutes of infusion [113]. DC activation was prevented, and by preventing migration to lymph nodes, there was a deficit in antigen presentation and CD4+ T cell production. Since this effect was observed 20 min after infusion, a circulating factor made by MSCs is likely responsible, possibly TSG-6 or galectin (discussed below). In addition to loss of antigen presentation by DCs, less IL-2 and TNF-α was observed, but no differences in IFN-γ production. Importantly, priming of MSCs by LPS exposure enhanced the effect (the inhibition of DC migration and activation). The impairment of DC migration and activation resulted in an inhibition of pathogenic antigen-specific T cells.

It has been shown repeatedly that MSCs limit the proliferation of stimulated CD4+ lymphocytes. *In vitro*, human MSCs impair expansion of stimulated CD4+ and CD8+ and impair IFN-γ synthesis by activate CD4+ and CD8+ cells [114]. Taken together, these findings support the observed immunomodulation effect of human MSCs in a xenogenic LPS-induced lung injury murine model which included enhanced survival, reduced pro-inflammatory mediators TNF-α, IL-1β, *etc.* [115].

Separation of GVHD and GVL: Recent work from Li *et al.* showed that chemokine receptor 7 (CCR7) was important for guiding MSCs to secondary lymphoid organs [116]. By enhancing migration of MSCs to secondary lymphoid organs, MSCs may enhance immune tolerance (reducing GVHD) while maintaining the GVL effect, although this has yet to be demonstrated clinically. As noted above, MSC infusion may prevent DC migration to peripheral lymph nodes [113] and thus slow DC-mediated T-cell expansion.

To summarize, MSCs from Wharton’s jelly, adipose tissue and bone marrow can potently suppress T-cell activation, and suppress both CD4+ and CD8+ T-cell proliferation induced by mitogen or allogeneic stimulation. Both soluble factors and direct contact are important for full effect of MSCs on immune cells. These studies did not consistently identify soluble factors involved; rather, they indicate a role for PGE2, IDO, COX 1 and 2, and other factors. The reason for these differences is unknown. Several studies indicate that differences exist between MSC sources, but the physiology that accounts for these differences is not currently understood. For example, does MSC proliferation or some other attribute limit MSC immune suppression [74]? MSCs from adipose, BM and WJ have similar *in vitro* and *in vivo* immune properties. The advantages of WJCs, e.g.; their lower immunogenicity, less immune activation and slower rejection compared to BM-MSCs, would not be apparent without direct comparisons (as was conducted in these studies). While some studies found that WJCs and adipose MSCs have equal or superior suppression of activated T-cell proliferation compared to BM-MSCs, in other studies the differences were less apparent. Finally, consistently, adipose and Wharton’s jelly MSCs have superior *in vitro* expansion properties compared to BM-MSCs.

## 14. Priming MSCs: Modification of MSC Properties for Regenerative Medicine

As mentioned above, MSCs’ immune properties—specifically their immunogenicity, ability to suppress T-cell activation, and immune suppression of activated T-cell and B-cell proliferation, can be licensed or primed. This suggests that the therapeutic effect of MSCs may be “tuned” to improve performance for a particular application. Several studies that address this hypothesis are discussed briefly below [28,74,78,86,87]. Cho *et al.* showed that exposure of WJCs to IFN-γ increases the expression of MHC class I and induces the expression of MHC class II [86]. This was accompanied by increases in the immunogenicity of WJCs in an allogeneic swine model. Similar and different findings were reported by Prasanna *et al.*, as was discussed above [74]. Again, the theme is that IFN-γ exposure modifies MSC effect on immune properties including expression of IDO, HLA class I and class II surface marker expression, *etc.* Tipnis *et al.* reported that IFN-γ caused WJCs to upregulate the expression of cell death ligand B7-H1, in addition to confirming that IFN-γ stimulates increased expression of IDO, and induces HLA class II expression [78]. Valencic *et al.* evaluated two variables: the priming effect of IFN-γ exposure on WJCs and the timing of lymphocyte exposure to WJCs [87]. They found that the timing of WJC priming was critical to reveal their immune suppressive effects on lymphocytes, and priming WJCs increased their immune suppressive action in both contact and non-contact settings. In contrast, if pre-stimulated lymphocytes were added to non-primed WJCs, the lymphocytes showed normal or enhanced proliferation. Deuse *et al.* examined the dose-dependent effects of IFN-γ on BM-MSCs and WJCs and found that higher levels of IFN-γ stimulation produce a stronger effect of WJCs on immune suppression [71].

The *in vitro* work suggests that primed MSCs would be more effective at treating chronic GVHD, where they are placed into an environment which will rapidly license them to begin immune suppression, which fits with animal model and human clinical observations [28,46]. It also suggests that un-primed MSCs given together with hematopoietic stem cells during allo-HCT would be ineffective at preventing GVHD, which again is supported by animal GVHD model work by Polchert *et al.* [28]. Moreover, such speculation might be retrospectively confirmed from clinical data. Additionally, the *in vitro* work suggests that IFN-γ-priming would improve MSCs’ therapeutic effect when given together with hematopoietic stem cells before GVHD has developed; this has been confirmed in a GVHD mouse model [28].

While Polchert’s work fits with *in vitro* work that indicates that IFN-γ priming will have beneficial effects in GVHD, primed MSCs have not yet been tested in clinical use. Currently, there is no reason to believe that primed MSCs would not be safe and effective for clinical use. In fact, the *in vitro* and animal model data suggest the primed MSCs would have more potent therapeutic effect than naïve MSCs. We further speculate that hindsight will clarify the target tissue effects reported for MSCs in GVHD [48] once the interactions of MSCs with Tregs, Th1, Th17 and Th2-cell subsets are resolved.

The issues with MSCs as a cellular therapy are the following: First, there remains concern about characterization of MSCs as a result of the ISCT consensus definition statement [117]. Efforts are underway to develop more refined methods for MSC characterization, production, and bioassays which would enable MSC optimization and clinical translation [114,118,119,120,121,122]. Second, MSCs are a heterogeneous population (discussed in [119]). Lacking standardized manufacturing methods and consensus on the identity of MSC subpopulations, identification of clinically relevant cells and optimization of clinical products is a concern. Third, it is assumed that there will be variability between samples that may result from differences between donors, e.g.; physiological differences, health status, donor age or sex. Additionally, MSCs may be isolated from different tissues from the same donor. These potentially confounding variables should be considered when developing reference materials [119]. Fourth, there is no uniform *in vitro* bioassay for MSC immune modulation to correlate with clinical response [114]. Thus, while human MSCs can be tested *in vitro*, or in xenogenic bioassays, no correlation between performance *in vitro* and clinical effect *in vivo* is established. These challenges exist for MSCs, but they have not posed a significant barrier for clinical testing: In CellTrials info blog site, Alexey Bersenev identified 90 MSC trials underway in 2013 worldwide and 1894 clinical trials are recruiting patients (found using the single search parameter: MSCs on the clinicaltrials.gov site 12 October 2014) [123].

## 15. Identification of MSC Stem Cell Subsets

As reviewed [124], nestin-positive cells have been shown as prospective markers for native MSC in mouse. Recently, PDGFR-α and CD51 have been identified as co-localizing on nestin-positive human MSCs [125]. These findings suggest a consistent theme across species and may enable follow up studies to confirm whether native human MSCs can be prospectively isolated and whether tissue-specific differences in native MSC populations exist. Others have suggested that clonal subsets of CD271+ cells contain most of the MSC CFU-F and may serve as an enriched population of native MSCs for clinical application [126]. Since we do not yet have an understanding of the *in vitro* expansion conditions needed to select and maintain native MSC populations, they have not been functionally characterized in terms of immune modulation properties.

## 16. Manufacturing MSCs for GVHD Treatment

In our previous review of this subject, we discussed licensing MSCs by INF-γ exposure to potentially improve their potency for GVHD [28,127]. Since then, MSCs have been shown to be even more plastic in their immune functions. For example, MSCs express toll-like receptors (TLR) 2, 3 and 4 [128,129,130]. Waterman *et al.* [130] neatly demonstrated two sharply contrasting MSC immune phenotypes that they called MSC type 1 and MSC type 2. MSC type 1 were primed using the specific TLR4 agonist LPS, and MSC type 2 were primed or licensed by the TLR3 agonist, poly (I:C). Stimulation by TLR4 resulted in the secretion of more pro-inflammatory factors, such as IL6, IL8, IL10, and TLR3-priming resulted in secretion of factors that were mostly immune suppressive, such as IL1RA, RANTES (CCL5), IP10 (CCL10) [130]. This observation fits with observations by Mastri *et al.* [131], who observed increased expression of IL6, IL10, IL11, HGF, LIF and TNF-α by MSCs after TLR3 priming using poly(I:C) and enhance therapeutic potency for cardiac repair. TLR activation of MSCs and IFN-γ priming of MSCs are likely needed to fully express the immune suppressive capabilities of MSCs. In summary, MSC potency as immune modulatory cells depends upon priming, and individual MSC clonal lines may use different modulation mechanisms [126]. In addition, it is likely that the expansion medium used for MSCs may impact their immune physiology [132] and tissue source of the MSC does, too [85,133,134].

## 17. Conclusions

In summary, MSCs appear to be safe and well-tolerated, and they offer a hope for treatment of steroid-refractory GVHD patients. The clinical outcomes reported to date have been encouraging; however, there is ample room for improvement. Most clinical trials have used BM-MSCs; adipose-derived MSCs were used in a few trials for GVHD, and WJCs have been tested clinically for GVHD in only two reported patients [58]. As discussed above, *in vitro* testing of MSCs suggests that off-the-shelf, unmatched cryopreserved MSCs derived from WJ may be a second generation of MSC-based cell therapy for GVHD. Finally, we must expand our understanding of the concept of priming MSCs since it improves effectiveness in an animal GVHD model and in pertinent *in vitro* assays. New information about MSC biology should be translated rapidly to clinical evaluation for safety and efficacy for therapy in steroid-resistant GVHD. Indeed, our center will soon begin enrollment of patients on a phase I clinical trial of *ex vivo* expanded WJCs in steroid-refractory aGVHD.
